# Synthesis and crystal structures of new chiral 3-amino-2*H*-azirines and the Pd com­plex of one of them

**DOI:** 10.1107/S2053229623001468

**Published:** 2023-02-23

**Authors:** Anthony Linden, Christoph B. Bucher, Ralf Gubler, José M. Villalgordo, Heinz Heimgartner

**Affiliations:** a Department of Chemistry, University of Zurich, Winterthurerstrasse 190, CH-8057 Zurich, Switzerland; University of Delaware, USA

**Keywords:** 3-amino-2*H*-azirines, azirine rings, crystal structure, diastereoisomers, Pd–azirine com­plex, organic synthesis

## Abstract

3-Amino-2*H*-azirines are potentially versatile building blocks in heterocyclic and peptide synthesis. Three new 3-amino-2*H*-azirines have been synthesized as racemates or mixtures of diastereoisomers in cases where another chiral residue is incorporated as the exocyclic amine. The crystal structures of two of them, an approximately 1:1 diastereoisomeric mixture of (2*R*)- and (2*S*)-2-ethyl-3-[(2*S*)-2-(1-meth­oxy-1,1-di­phenyl­meth­yl)pyrrolidin-1-yl]-2-methyl-2*H*-azirine, C_23_H_28_N_2_O, **11**, and 2-benzyl-3-(*N*-methyl-*N*-phenyl­amino)-2-phenyl-2*H*-azirine, C_22_H_20_N_2_, **12**, and the third as its diastereoisomeric *trans*-PdCl_2_ com­plex, *trans*-di­chlorido­[(2*R*)-2-ethyl-2-methyl-3-(*X*)-2*H*-azirine][(2*S*)-2-ethyl-2-methyl-3-(*X*)-2*H*-azirine]palladium(II), where *X* = *N*-{[(1*S*,2*S*,5*S*)-6,6-di­methylbi­cyclo­[3.1.1]heptan-2-yl]meth­yl}-*N*-phenyl­amino, [PdCl_2_(C_21_H_30_N_2_)_2_], **14**, have been determined and the geometries of the azirine rings com­pared with those of 11 other 3-amino-2*H*-azirine structures reported in the literature. Most notable is the very long formal N—C single bond, which is, with one exception, around 1.57 Å. Each com­pound has crystallized in a chiral space group. The Pd atom in the *trans*-PdCl_2_ com­plex is coordinated by one of each of the pair of diastereoisomers, while both of the diastereoisomers share the same crystallographic site in the structure of **11**; this property thereby manifesting itself as disorder. The chosen crystal of **12** is either an inversion twin or com­posed of a pure enanti­omorph, but this could not be established specifically.

## Abstract 2

Three new 3-amino-2*H*-azirine derivatives have been synthesized as racemates or mixtures of diastereoisomers. Comparison of their crystal structures with those of the 11 other 3-amino-2*H*-azirines in the literature reveal that the formal N—C single bond in the azirine ring is consistently long at around 1.57 Å.

## Introduction

Since the first synthesis of 3-amino-2*H*-azirines (Rens & Ghosez, 1970 ▸), the chemistry of these three-membered cyclic amidines has been studied intensively (Heimgartner, 1979 ▸, 1981 ▸, 1986 ▸, 1991 ▸; Eremeev & Piskunova, 1990 ▸). They have been found to be versatile building blocks in heterocyclic and peptide synthesis. In com­parison with the better known 3-aryl-2*H*-azirines (three-mem­bered cyclic imines), the 3-amino derivatives are stronger bases and more reactive nucleophiles. For example, 3-phenyl-2*H*-azirine reacts with carb­oxy­lic acids in refluxing benzene to give the corresponding *N*-phenacyl­car­box­amides (Sato *et al.*, 1967 ▸; Black & Doyle, 1978 ▸), and the reaction of 2,2-dimethyl-3-phenyl-2*H*-azirine with mercapto­acetic acid was performed in acetone at 343 K for 15 h yielding *N*-(1,1-dimethyl-2-oxo-2-phenyl­eth­yl)-2-mercaptoacetamide (Él’kinson & Eremeev, 1986 ▸). Only 3-alkyl-2*H*-azirine-2-phos­phine oxides exhibited a higher reactivity; a slow reaction with carb­oxy­lic acids in tetra­hydro­furan (THF) occurs already at room temperature within 1–4 days (Palacios *et al.*, 2002 ▸). On the other hand, *N*,*N*-disubstituted 3-amino-2*H*-azirines of type **1** react with carb­oxy­lic acids (Vittorelli *et al.*, 1974 ▸; Obrecht & Heimgartner, 1983 ▸) and N-protected amino acids (Obrecht & Heimgartner, 1987 ▸; Wipf & Heimgartner, 1988 ▸; Dan­necker-Dörig *et al.*, 2011 ▸) at 273–298 K within a few minutes to give products of type **2** (Scheme 1). The analogous reaction of 3-amino-2-phenyl­carbamoyl-2*H*-azirine with acetic acid in ace­tone was carried out at 323 K within 1 h (Eremeev *et al.*, 1985 ▸). 
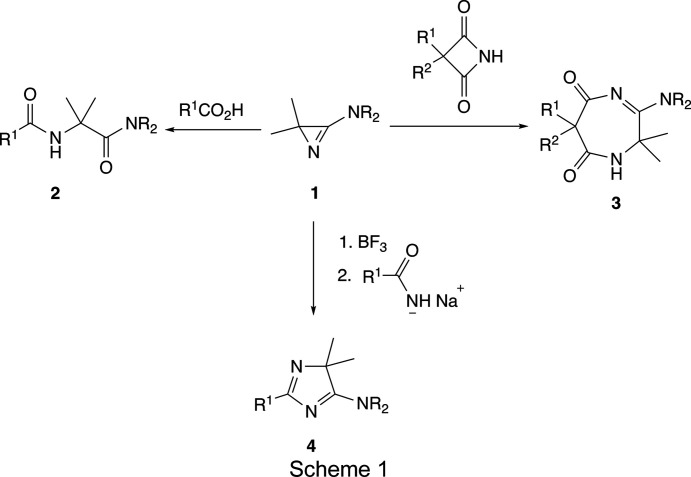


Furthermore, 3-amino-2*H*-azirines, **1**, react spontaneously with NH-acidic heterocycles if their p*K_a_* value is less than 8 (Chaloupka *et al.*, 1977 ▸; Scholl *et al.*, 1978 ▸). For example, the reaction with 3,3-disubstituted azetidine-2,4-diones (malon­imides) in 2-propanol at room temperature yields 1,4-diazepine derivatives, **3** (Scheme 1). In all of these reactions, **1** has to be activated by protonation to enable the addition of the nucleophilic com­pound. On the other hand, reactions of **1** with non-acidic N-nucleophiles, such as primary amino com­pounds (Hugener & Heimgartner, 1995 ▸) or sodium amidates (Arnhold *et al.*, 1995 ▸), can be performed *via* BF_3_ catalysis. In the latter case, 4,4-di­substituted 5-amino-4*H*-imidazoles, **4**, are formed (Scheme 1); the reaction mechanism is explained by the initial com­plexation of the ring N atom of **1** with BF_3_. Similarly, the ZnCl_2_-catalyzed reaction of 3-aryl-2*H*-azirines with benzimidates has been elaborated as an efficient preparation of imidazoles (Shi *et al.*, 2018 ▸). 
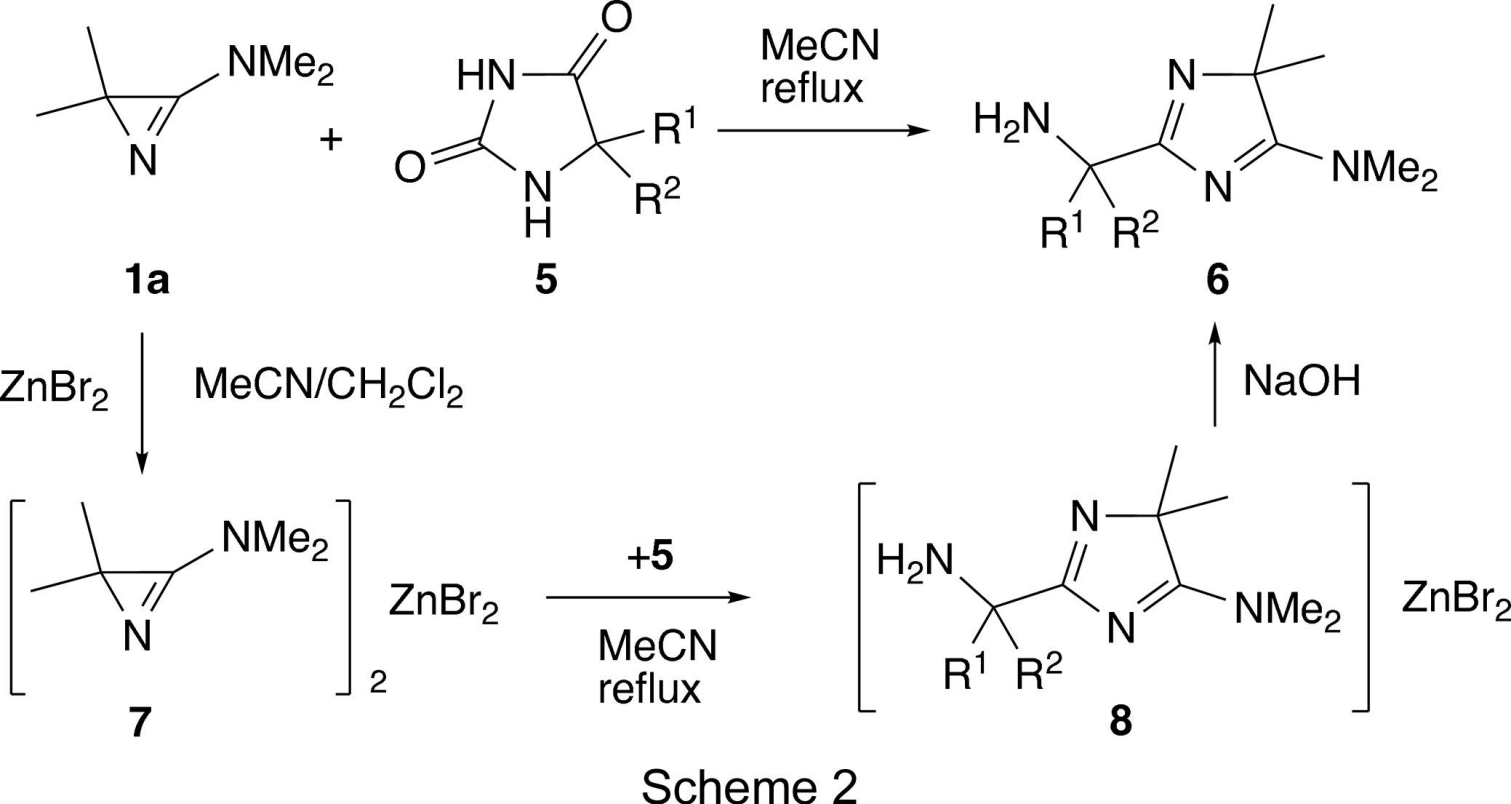


Based on these results, we expected that reactions of **1** with nucleophiles may also be catalyzed by com­plexation of **1** with ZnBr_2_ or PdCl_2_. Corresponding com­plexes of 3-amino-2*H*-azirines **1** are known (Hassner *et al.*, 1978 ▸; Dietliker *et al.*, 1978 ▸; Dos Santos Filho *et al.*, 1983 ▸; Heimgartner, 1991 ▸; Villalgordo & Heimgartner, 1993 ▸). Unfortunately, attempts to catalyze the reaction of **1a** with imidazolidine-2,4-diones (hydantoins), **5**, by using ZnBr_2_ (Scheme 2) were only mildly successful (Schläpfer-Dähler *et al.*, 1992 ▸). Whereas the formation of the 4*H*-imidazole derivatives, **6**, from **1a** and **5** was achieved in refluxing aceto­nitrile within two days, the reaction of the azirine com­plex **7** with **5** was com­plete after 14–24 h, and after decom­plexation of the 4*H*-imidazole com­plexes, **8**, by treatment with NaOH, com­pounds **6** were obtained in slightly increased yields. 
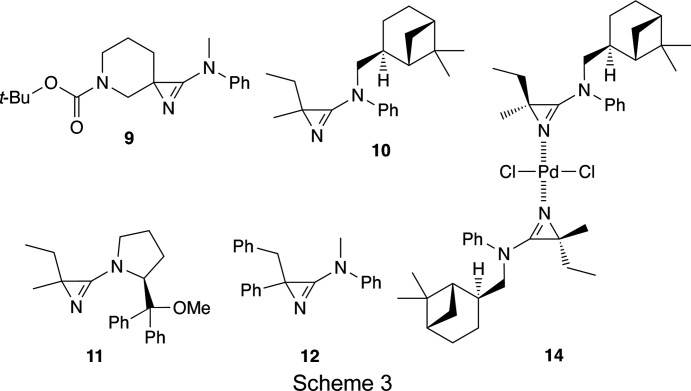


On the other hand, we successfully used the com­plexation of the hetero­spiro­cyclic 3-amino-2*H*-azirine, **9**, with PdCl_2_ for the chromatographic purification of this com­pound as a race­mate (Villalgordo & Heimgartner, 1993 ▸). Because 3-am­ino-2*H*-azirines with two different substituents at the alkyl atom, C2, of the azirine ring, *e.g.* **10**–**12**, are useful building blocks for chiral α,α-disubstituted α-amino acids, the separation of the diastereoisomers or enanti­omers is an important issue (Bucher *et al.*, 1995 ▸, 1996 ▸, 2020 ▸; Brun *et al.*, 2001 ▸, 2002 ▸). Therefore, we synthesized the azirines **10**–**12** with the aim of separating the stereoisomers after their direct crystallization or crystallization of their PdCl_2_ com­plexes (Scheme 3).

## Experimental

### Synthesis and crystallization

The 3-amino-2*H*-azirines **10**–**12** were prepared according to previously described syntheses. In the case of **10**, sequential treatment of 1 g (3.19 mmol) of a diastereomeric mixture of the corresponding 2-methyl­butyric acid amide, **13**, bearing the chiral residue derived from (−)-*trans*-myrtanol, in dry THF (15 ml) with lithium diiso­propyl­amide (LDA), di­phenyl­phosphoryl chloride (DPPCl) and NaN_3_ in DMF (Scheme 4; Villalgordo, 1992 ▸; *cf.* Villalgordo & Heimgartner, 1993 ▸) led to the desired product. Chromatographic work-up on SiO_2_ (hex­ane–AcOEt, 9:1 *v*/*v*) gave 712 mg (72%) of **10** as a mix­ture of diastereoisomers as a slightly yellow oil. To a well-stirred suspension of 156 mg (0.654 mmol) PdCl_2_ in dry aceto­nitrile (MeCN, 1.5 ml) at 273 K was added a solution of 200 mg (0.654 mmol) of azirine **10** in MeCN (0.5 ml). After stirring for 10 h, the solvent was partially evaporated and the residue was filtered through a short column of SiO_2_ (hexa­ne–ethyl acetate, 9:1 *v*/*v*). Evaporation of the solvents gave 475 mg (92%) of the Pd com­plex, **14**, as a red–orange solid. Recrystallization from MeCN by slow evaporation of the 
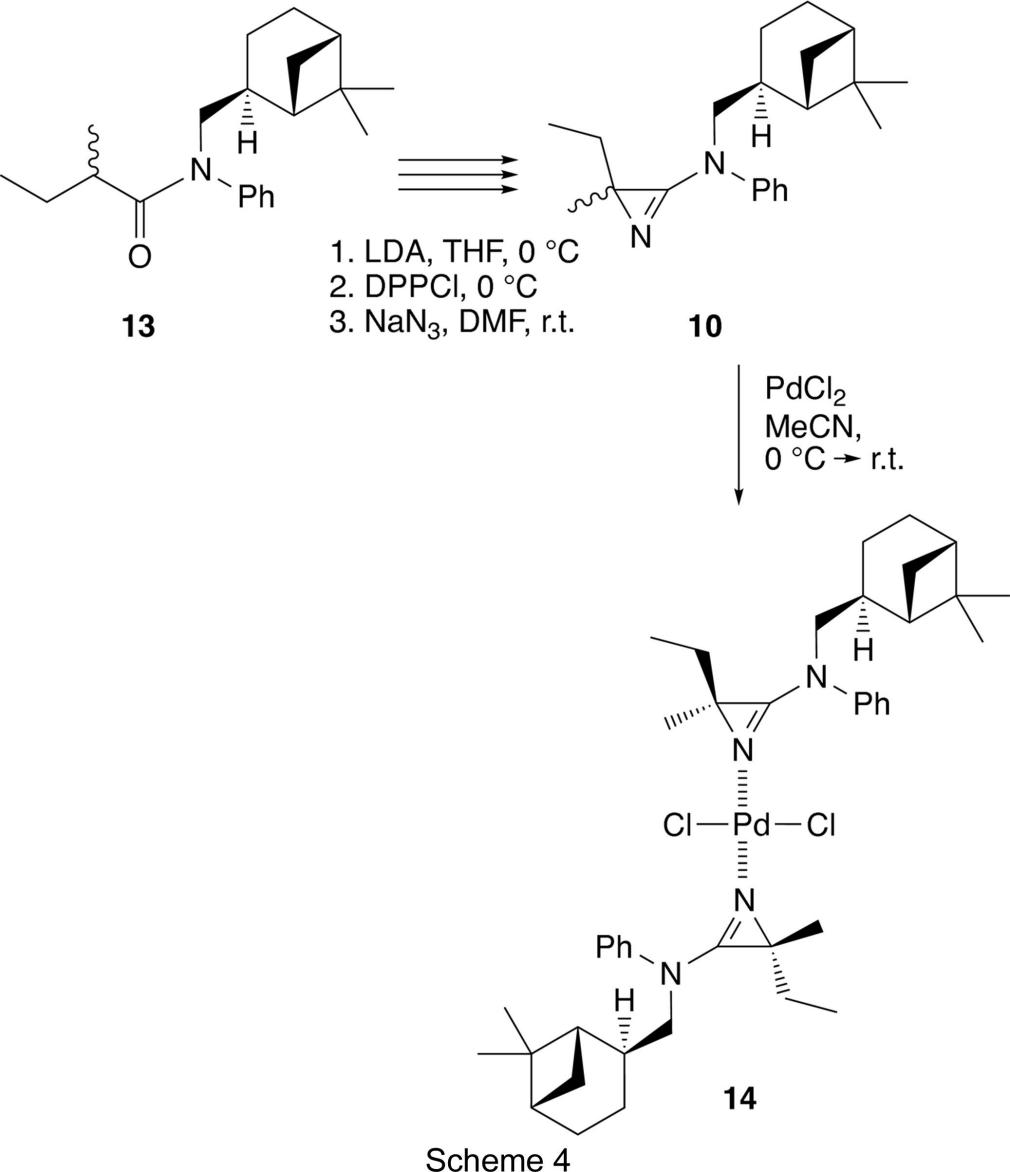
 solvent yielded orange crystals of suitable quality for crystal structure analysis. The crystal structure of **14** revealed that one of each of the diastereoisomers of **10** were coordinated to the Pd centre to give a mol­ecule with the absolute stereochemistry shown in Scheme 4. 
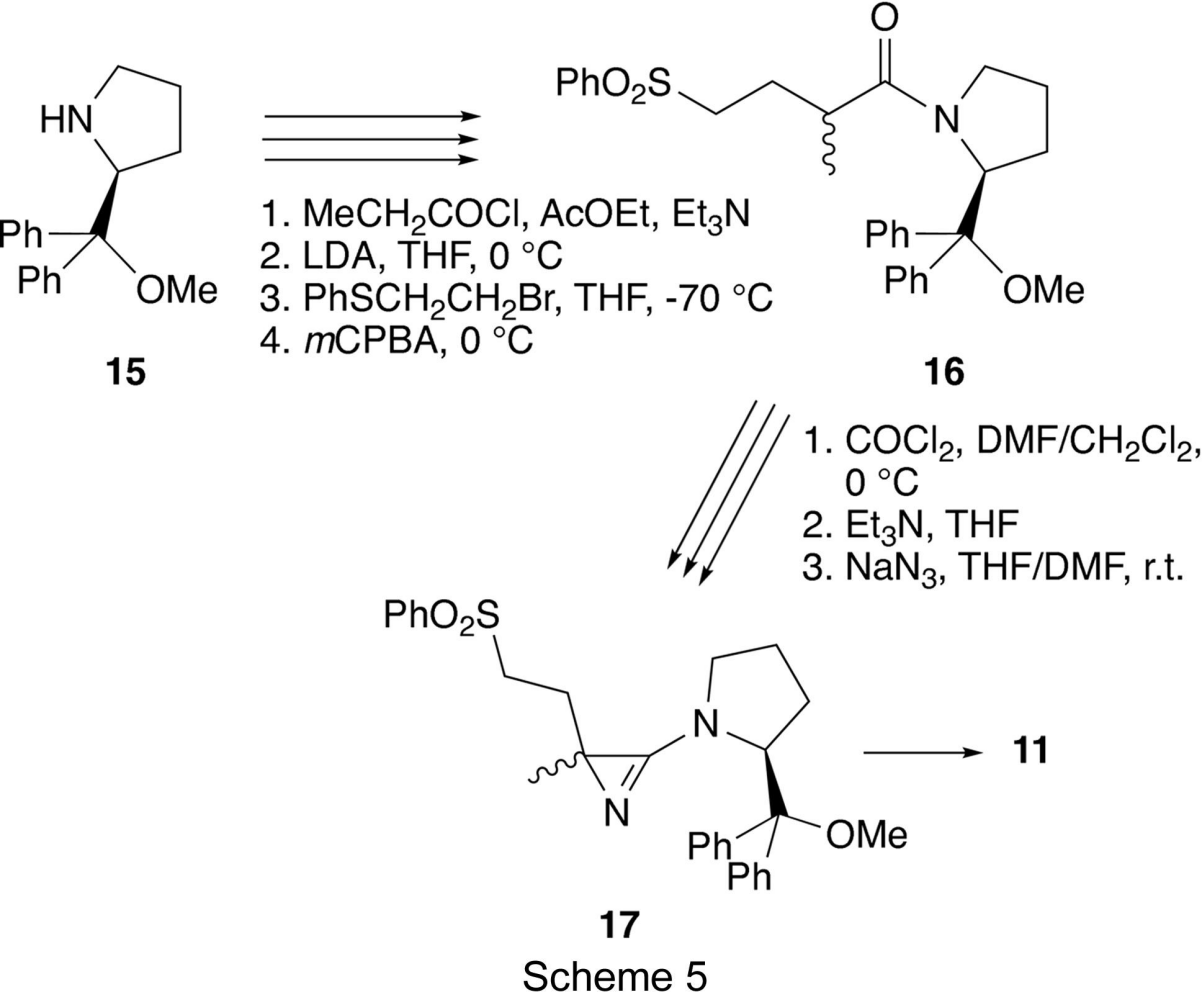


Starting with the known (*S*)-pyrrolidine derivative **15** (Enders *et al.*, 1988 ▸), the azirine **17** was prepared following pro­cedures described earlier (Scheme 5; Bucher, 1996 ▸; *cf*. Bucher & Heimgartner, 1996 ▸). Whereas the precursor **16** was obtained in good yield (84%) as a mixture of diastereoisomers, the standard transformation to the amino­azirine led to a *ca* 2:1 mixture of the diastereomeric azirines **17** in only 10% yield. The electrolytical removal of the phenyl­sulfonyl group (–2.1 V, EtOH, Me_4_NCl, 278 K; *cf*. Bucher & Heimgartner, 1996 ▸) gave only a few crystals of the desired azirine **11**, which were recrystallized from MeOH/Et_2_O, yielding crystals suitable for crystal structure determination. 
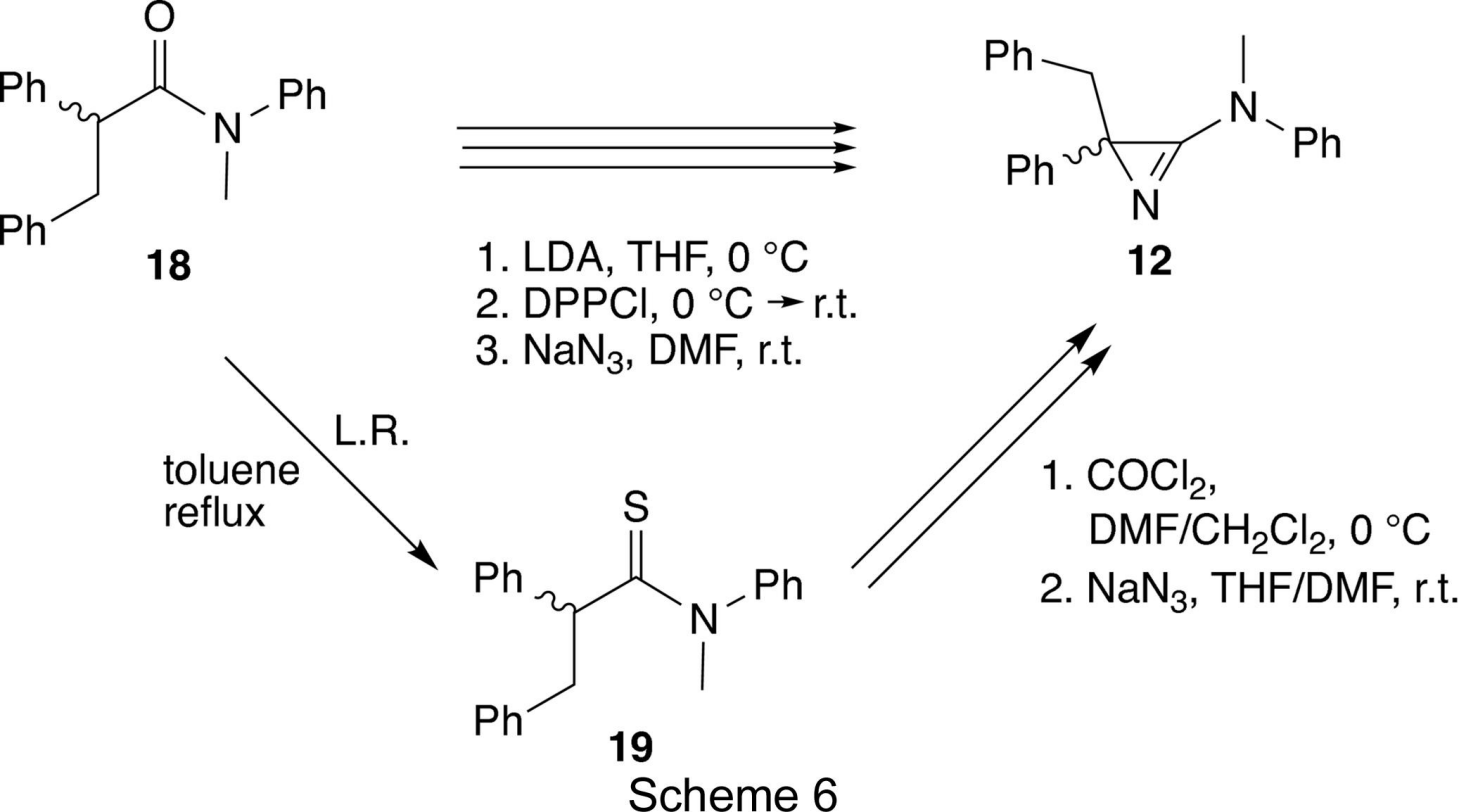


3-Amino-2-benzyl-2-phenyl-2*H*-azirine **12** was synthesized either from the amide **18** (*cf*. Villalgordo & Heimgartner, 1993 ▸) or the thio­amide **19** (*cf*. Bucher *et al.*, 1995 ▸; Brun *et al.*, 2002 ▸), respectively (Scheme 6). In the first case, starting with 2.50 g (7.9 mmol) of **18**, azirine **12** was obtained in 70% yield (1.74 g) as a slightly yellow oil, which solidified under high vacuum (Gubler, 1996 ▸). In the second approach, the amide **18** was transformed into the thio­amide **19** in 94% yield, and 19.20 g (57.9 mmol) of the latter were treated with phosgene in DMF/CH_2_Cl_2_ and then with sodium azide in THF/DMF to give 11.05 g (61%) of azirine **12** as a yellowish solid. Recrystallization of the azirine **12** from Et_2_O/hexane yielded colourless crystals of a single enanti­omer suitable for a crystal structure analysis.

### Analytical and spectroscopic data

Compound **10** (mixture of diastereoisomers): slightly yellow oil; IR (CHCl_3_): 2970 (*s*), 2920 (*s*), 2870 (*m*), 1745 (*s*), 1600 (*s*), 1500 (*s*), 1455 (*m*), 1375 (*m*), 1365 (*m*), 1290 (*m*), 1240 (*m*), 1185 (*s*), 1155 (*m*), 1100 (*m*), 965 (*m*), 690 (*m*) cm^−1^; ^1^H NMR (CDCl_3_): δ 7.4–7.0 (*m*, 5 arom. H), 3.72 (*br s*, CH_2_N), 2.53 (*br s*, 1H), 2.15–2.05 (*m*, 1H), 1.9–1.55 (*m*, 9H), 1.39, 1.16 (2*s*, 2 Me), 0.74 (*br s*, Me), 0.73 (*s*, Me); ^13^C NMR (CDCl_3_): δ 166.6 (*s*, C=N), 150.3, 141.7 (2*s*, 1 arom. C), 129.6, 129.1, 125.3, 123.1, 119.9, 117.3 (6*d*, 5 arom. CH), 63.4, 51.4 (2*t*, CH_2_N), 42.6 (*d*, CH), 40.5 (*q*, Me), 38.9 (*s*, Me_2_ *C*), 32.5 (*d*, CH), 30.0, 29.9 (2*t*, CH_2_), 26.4 (*d*, CH), 23.7, 23.3 (2*t*, 2 CH_2_), 19.7 (*q*, Me), 19.0, 18.9 (2*t*, CH_2_), 9.6 (*q*, Me); CI–MS: 311 (100, [*M* + 1]^+^). Compound **14**: orange solid; m.p. 411–413 K; IR (KBr): 2910 (*s*), 1800 (*s*), 1595 (*s*), 1495 (*s*), 1460 (*m*), 1380 (*m*), 1365 (*m*), 1230 (*m*), 1215 (*m*), 1200 (*m*), 1155 (*m*), 1080 (*m*), 1065 (*m*), 760 (*m*), 690 (*s*) cm^−1^; ^1^H NMR (DMSO-*d*_6_): δ 7.5–7.15 (*m*, 10 arom. H), 4.45–4.25 (*m*, 1H), 4.2–3.95 (*m*, 2H), 3.66 (*br s*, 2H), 2.3–1.9 (*m*, 4H), 1.85–1.45 (*m*, 15H), 1.4–1.25 (*m*, 8H), 1.15–1.05 (*m*, 7H), 0.75–0.55 (*m*, 11H); ^13^C NMR (DMSO-*d*_6_): δ 164.2, 164.0 (2*s*, 2 C=N), 140.3, 140.2 (2*s*, 2 arom. C), 130.1, 129.7, 125.7, 119.9, 119.7, 119.5 (6*d*, 10 arom. CH), 52.7, 52.5 (2*t*, 2 CH_2_N), 49.2, 49.1 (2*s*, 2 C), 40.7, 40.6 (2*d*, 2 CH), 32.5, 32.1 (2*d*, 2 CH), 29.4, 29.3, 28.4, 28.3 (4*t*, 4 CH_2_), 26.4, 26.3 (2*q*, 2 Me), 23.6, 23.5 (2*t*, 2 CH_2_), 22.8, 22.6 (2*d*, 2 CH), 19.8, 19.6 (2*q*, 2 Me), 18.4, 18.2 (2*t*, 2 CH_2_), 9.6, 9.2 (2*q*, 2 Me).

Compound **16** (mixture of diastereoisomers): colourless oil; IR (CHCl_3_): 3000 (*m*), 1625 (*s*), 1495 (*w*), 1465 (*m*), 1450 (*s*), 1310 (*s*), 1150 (*s*), 1090 (*s*), 1075 (*s*), 705 (*s*), 690 (*m*) cm^−1^; ^1^H NMR (CDCl_3_) (2 diastereoisomers, 1 rotamer): δ 7.95–7.85, 7.7–7.55, 7.45–7.35 (3*m*, 15 arom. H), 5.55, 5.41, 5.00 (3*d*, 1H), 3.7–3.65, 3.6–3.5 (2*m*, 1H), 3.3–2.95 (*m*, 2.5H), 2.9–2.8 (*m* with 3*s* at 2.89, 2.86, and 2.83, 1H and MeO), 2.7–2.6 (*m*, 0.5H), 2.2–1.85 (*m*, 4H), 1.55–1.35 (*m*, 1H), 1.2–1.05 (*m* with 2*d* at 1.18, 1.06, 2.5H), 1.1–0.95 (*m*, 1H), 0.85–0.75 (*m*, 0.5 H); CI–MS: 492 ([*M* + H]^+^), 460 ([*M* − MeO]^+^). Compound **17** (mixture of diastereoisomers): colourless solid; IR (KBr): 2950 (*m*, broad), 1765 (*s*), 1450 (*s*), 1310 (*s*), 1150 (*s*), 1085 (*m*), 1070 (*m*), 760 (*m*), 705 (*m*), 690 (*m*) cm^−1^; ESI–MS: 511 ([*M* + Na]^+^), 489 ([*M* + H]^+^). Compound **11** (mixture of diastereoisomers): colourless crystals.

Compound **12** (mixture of enanti­omers): yellowish solid; m.p. 345–348 K; IR (CHCl_3_): 2985 (*m*), 1760 (*s*, broad), 1600 (*s*), 1498 (*s*), 1452 (*m*), 1390 (*m*), 1326 (*m*), 1283 (*m*), 1110 (*m*), 1075 (*m*), 696 (*m*); ^1^H NMR (DMSO-*d*_6_): δ 7.4–7.05 (*m*, 15 arom. H), 3.61, 3.49 (AB, *J*_AB_ = 15.0, PhCH_2_), 3.26 (*s*, MeN); ^13^C NMR (DMSO-*d*_6_): δ 159.6 (*s*, C=N), 143.4, 142.7, 138.0 (3*s*, 3 arom. C), 130.1, 129.5, 128.5, 128.1, 126.8, 126.7, 126.3, 123.6, 117.6 (9*d*, 15 arom. CH), 41.3 (*s*, C2), 40.3 (*t*, PhCH_2_), 36.0 (*q*, CH_3_N); EI–MS: 312 (*M* ^+**.**^), 297 ([*M* – CH_3_]^+^), 221 ([*M* – C_7_H_7_]^+^), 206, 178, 118, 103, 91, 77.

### Refinement

Crystal data, data collection and structure refinement details are summarized in Table 1 ▸. For each structure, the methyl H atoms were constrained to an ideal geometry (C—H = 0.98 Å), with *U*_iso_(H) = 1.5*U*_eq_(C), but were allowed to rotate freely about the C—C bonds. All other H atoms were placed in geometrically idealized positions and constrained to ride on their parent atoms with C—H distances of 0.95 (aromatic), 0.99 (methyl­ene) or 1.00 Å (methine) and with *U*_iso_(H) = 1.2*U*_eq_(C).

**Figure 1:**
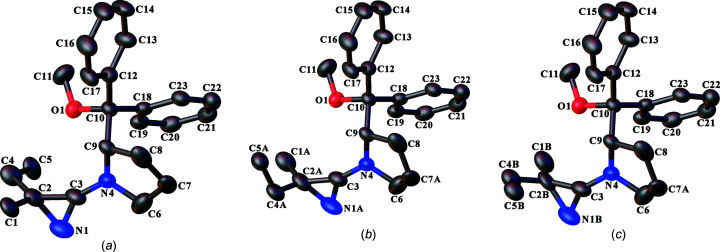
Views and atom-labelling schemes of the individual disordered com­ponents in the mol­ecular structure of **11**, showing (*a*) the disorder com­ponent com­posed of the 2*R* diastereoisomer, and (*b*) the major and (*c*) the minor disordered conformations in the com­ponent com­posed of the 2*S* diastereo­isomer. Displacement ellipsoids are drawn at the 50% probability level. H atoms have been omitted for clarity. An overlay of all three disorder com­ponents is presented in the supporting information (Fig. S1).

The mol­ecule in the crystal structure of com­pound **11** is disordered in two regions. Atom C7 of the five-membered ring occupies two positions which represent alternate envelope conformations of the ring; the site-occupation factor of the major conformer refined to 0.619 (18). In addition, the azirine ring and its C2-ethyl and methyl substituents required three sets of positions to adequately model the arrangement. These positions indicate that the 2*R* and 2*S* diastereoisomers have crystallized at the same crystallographic site in the crystal and that the 2*S* diastereoisomer is further disordered over two conformations. The site occupation of the 2*R* con­figuration at atom C2 refined to 0.432 (3), while the site-occupation factors for the two conformations of the 2*S* diastereoisomer refined to 0.305 (3) and 0.263 (3) for the conformations containing atoms with *A* and *B* suffixes, respectively, in their labels (Fig. 1 ▸). Target bond-length restraints were applied to the disordered atoms. In addition, similarity restraints were applied to the chemically equivalent bond lengths and angles involving all disordered atoms, while neighbouring atoms within and between each arrangement of the disordered groups were restrained to have similar atomic displacement parameters.

In the structure of **14**, the chiral residue derived from (−)-*trans*-myrtanol in each ligand is conformationally disordered. Two sets of positions were defined for the atoms of each disordered residue and the site-occupation factors of the major conformations of these groups refined to 0.621 (11) and 0.675 (9) for the ligands containing atoms N1 and N21, respectively. Similarity restraints were applied to the chemically equivalent bond lengths involving all disordered C atoms, while neighbouring atoms within and between each conformation of the disordered groups were restrained to have similar atomic displacement parameters.

## Results and discussion

**Figure 2:**
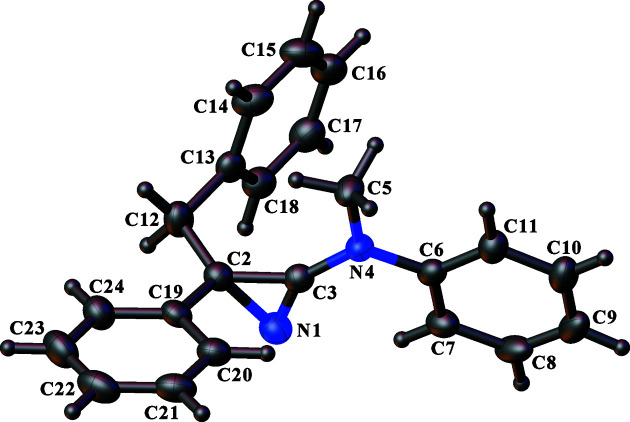
View of the mol­ecule of **12**, showing the atom-labelling scheme. Displacement ellipsoids are drawn at the 50% probability level. H atoms are represented by circles of arbitrary size.

**Figure 3:**
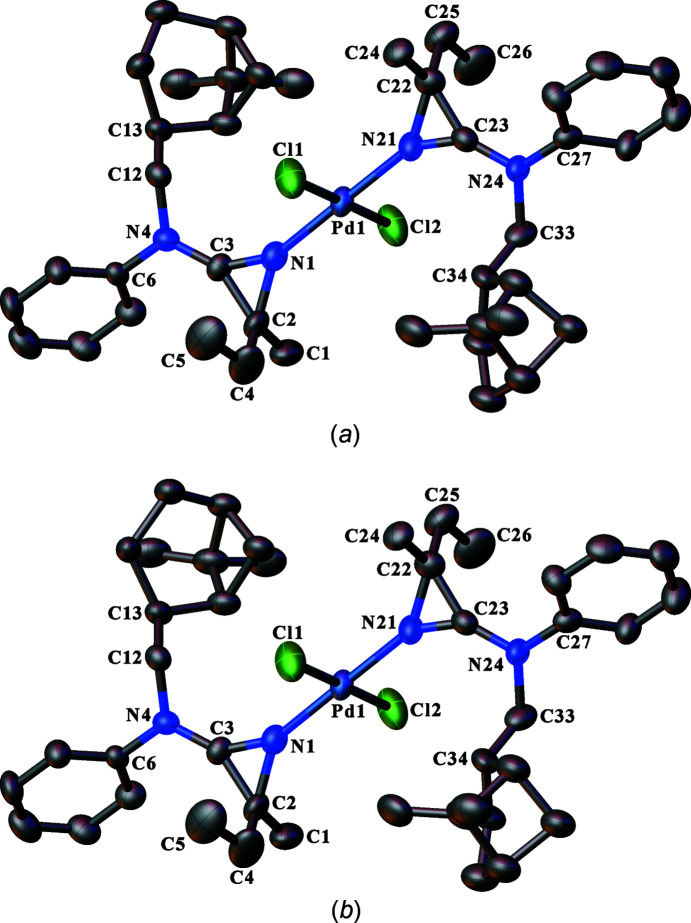
Views of the (*a*) major and (*b*) minor disorder com­ponents of the mol­ecule of **14**, showing the atom-labelling schemes. Displacement ellipsoids are drawn at the 50% probability level. H atoms have been omitted for clarity. An overlay of the two disorder com­ponents is presented in the supporting information (Fig. S2).

The syntheses described in the *Introduction* include nonstereo­specific reactions during the azirine ring formation to give 3-amino-2*H*-azirines. Therefore, the products are expected to be either racemic mixtures or, when another residue in the mol­ecule contains one or more invariant stereogenic centres, mixtures of diastereoisomers. The three crystal structures described here are of crystals obtained from the products **11** (Fig. 1 ▸), **12** (Fig. 2 ▸) and the PdCl_2_ com­plex with **10** (**14**; Fig. 3 ▸). The chosen crystal in each case had crystallized in a chiral space group, which is a necessity for **11** and **14**, because these mol­ecules contain invariant chiral exocyclic amine residues derived from the known (*S*)-pyrrolidine derivative, **15**, and (−)-*trans*-myrtanol {*i.e*. [(1*S*,2*S*,5*S*)-6,6-di­methylbi­cyclo­[3.1.1]heptan-2-yl]methanol}, respectively. The absolute structure chosen when refining the models for **11** and **14** was thus aligned to match the chirality of the known chiral residues. In the case of **14**, the strong anomalous scattering power im­parted by the Pd and Cl atoms allowed the absolute con­fig­uration of all stereogenic centres to be confirmed confidently from the diffraction experiment by refinement of the absolute structure parameter (Flack & Bernardinelli, 1999 ▸, 2000 ▸), which converged to a value of −0.02 (2). The absolute structure of **11** could not be determined independently from the diffraction experiment on account of the weak anomalous scattering power of the com­pound with the available Mo *K*α X-ray radiation (the work was carried out in the early 1990s when it was not common to use Cu *K*α radiation routinely).

In contrast, com­pound **12** only contains a single stereogenic centre, which is at atom C2 of the azirine ring, so a racemic mixture could conceivably have crystallized in an achiral space group. Given that the synthesis of com­pound **12** most likely produced a racemic mixture of the product and not a single enanti­omer, the fact that com­pound **12** crystallized in a chiral space group indicates that either a single enanti­omer has crystallized in a spontaneous resolution process, or the crystal is an inversion twin and therefore a racemic mixture or another ratio (solid solution) of enanti­omers. For the same reasons as given above for com­pound **11**, the absolute structure of **12** could not be determined. Therefore, the presence of a specific enanti­omer or even an inversion twin could not be established and the con­figuration of the mol­ecule defined in the refinement model and depicted in Fig. 2 ▸ was chosen arbitrarily.

The unique mol­ecule in the crystal structure of com­pound **11** is disordered in two regions (Fig. 1 ▸). The five-membered pyrrolidine ring has two distorted envelope conformations, while the azirine ring and its C2-ethyl and methyl substituents are disordered over three arrangements. The disorder model indicates that the 2*R* and 2*S* diastereoisomers are present in the crystal and are distributed randomly at the same crystallographic site. There is a slight excess of the 2*S* diastereo­isomer, which is disordered additionally over two con­form­ations (see Section 2.3 for more details).

The crystal structure of com­pound **14** reveals one symmetry-unique *trans*-PdCl_2_ *L* ^1^ *L* ^2^ com­plex mol­ecule, where *L* ^1^ and *L* ^2^ are diastereoisomers of product **10**, which coordinate to the metal *via* their azirine ring N atom (Fig. 3 ▸). The diastereoisomers are the 2*S* and 2*R* species which result from inter­change of the positions of the ethyl and methyl substituents at atom C2 of the azirine ring, while the con­figuration of the chiral residue derived from (−)-*trans*-myrtanol remains con­stant. It is perhaps remarkable that the Pd com­plex con­tains one of each of the pair of diastereoisomers, as conceivably the com­plex could consist of two of the same di­as­tereo­isomer or a nonstoichiometric ratio of the two diastereoisomers, which would manifest itself in the same sort of disorder of the ethyl and methyl substitution site that was observed for **11**, as described above. In the structure of **14**, the chiral residue derived from (−)-*trans*-myrtanol in each ligand is conformationally disordered (Fig. 3 ▸), but this has no consequence for the unique absolute con­figuration of the residue. The coordination geometry around the Pd atom is square planar, as usual, and the coordination geometry is listed in Table 2 ▸.

Reports of crystal structures of 3-amino-2*H*-azirines are quite rare. The Cambridge Structural Database (CSD; Version 5.43 with November 2022 updates; Groom *et al.*, 2016 ▸) lists only 11 structures, of which seven have been reported by the Heimgartner group (Villalgordo & Heimgartner, 1992 ▸; Bucher & Heimgartner, 1996 ▸; Brun *et al.*, 2001 ▸) and the remaining four were reported by Galloy *et al.* (1974 ▸, 1980 ▸), Piskunova *et al.* (1993 ▸) and Peters *et al.* (2000 ▸). The geometry of the azirine ring (Table 3 ▸) generally shows little variation across all of these structures. Possibly the most remarkable feature is the very long N—C single bond, which is, with one exception, always around 1.57 Å [mean 1.572 (5) Å for 10 structures], com­pared with N—C distances closer to 1.47 Å usually found for simple imines. This contrasts with the shorter formal C—C single bond with a mean length of 1.437 (7) Å. The short formal C—N single bond to the exocyclic N atom, with a mean value of 1.333 (12) Å, is likely a consequence of electron-pair delocalization between the exocyclic N atom and the ring N=C bond; Galloy *et al.* (1974 ▸) described this as the consequence of a contribution from a polar mesomeric form. The biggest ring geometry outlier amongst the 11 structures men­tioned above is in the structure of 3-di­methyl­amino-2-di­methyl­carbamoyl-2-phen­oxy-2*H*-azirine (3-phen­oxy-3-di­methyl­carbamoyldi­methyl­amino-2-azirine) (Galloy *et al.*, 1974 ▸), in which, in particular, the ring N—C single bond of 1.49 Å is significantly shorter than in the other structures. This might result from the inductive electron-withdrawal properties of the O atom in the phenoxy substituent at the azirine ring *sp* ^3^-hybridized C atom, whereas all other structures have C atoms as the first atom of each substituent. The three new crystal structures reported here are no exception, notwithstanding the potential low accuracy for the disordered azirine ring in **11** because of the restraints applied while modelling the disorder; see Section 2.3. The coordination of the azirine rings *via* their N atom to the Pd atom in com­plex **14** also appears to influence very slightly the geometry of the azirine ring to give marginally shorter N—C and longer C—C single bonds, respectively (Table 3 ▸). This is perhaps unsurprising given the change in the electronic properties as a result of the coordination.

## Conclusion

The 3-amino-2*H*-azirines **10**–**12** were synthesized with the aim of separating the stereoisomers after their direct crystallization or crystallization of their PdCl_2_ com­plexes, as exemplified by com­plex **14**, which incorporates com­pound **10** as ligands. Unfortunately, this objective was not achieved, as the crystal structures of **11** and **14** revealed the presence of a diastereoisomeric mixture of the azirines in the crystals, and the crystal structure of **12** was inconclusive as to whether the chosen crystal was enanti­omerically pure or also a racemic mixture that had crystallized as an inversion twin. Nonetheless, the study has added to the small number of recorded crystal structures of amino­azirines with their unusually long formal ring N—C single bonds.

## Supplementary Material

Crystal structure: contains datablock(s) 11, 12, 14, global. DOI: 10.1107/S2053229623001468/yp3228sup1.cif

Structure factors: contains datablock(s) 11. DOI: 10.1107/S2053229623001468/yp322811sup2.hkl

Supporting information file. DOI: 10.1107/S2053229623001468/yp322811sup5.cml

Structure factors: contains datablock(s) 12. DOI: 10.1107/S2053229623001468/yp322812sup3.hkl

Supporting information file. DOI: 10.1107/S2053229623001468/yp322812sup6.cml

Structure factors: contains datablock(s) 14. DOI: 10.1107/S2053229623001468/yp322814sup4.hkl

Supporting information file. DOI: 10.1107/S2053229623001468/yp322811sup5.cml

Supporting information file. DOI: 10.1107/S2053229623001468/yp322812sup6.cml

Additional figures. DOI: 10.1107/S2053229623001468/yp3228sup7.pdf

CCDC references: 2243434, 2243435, 2243436

## Figures and Tables

**Table 1:** Experimental details Experiments were carried out at 173 K with Mo *K*α radiation using a Rigaku AFC-5R diffractometer. H-atom parameters were constrained.

	**11**	**12**	**14**
Crystal data			
Chemical formula	C_23_H_28_N_2_O	C_22_H_20_N_2_	[PdCl_2_(C_21_H_30_N_2_)_2_]
*M*_r_	348.47	312.40	798.24
Crystal system, space group	Monoclinic, *P*2_1_	Orthorhombic, *P*2_1_2_1_2_1_	Triclinic, *P*1
*a*, *b*, *c* (Å)	7.792 (4), 14.462 (6), 9.113 (3)	10.642 (2), 15.8762 (18), 10.273 (2)	9.070 (2), 10.504 (6), 11.756 (2)
α, β, γ (°)	90, 105.87 (3), 90	90, 90, 90	80.14 (3), 76.054 (19), 73.67 (2)
*V* (Å^3^)	987.8 (8)	1735.7 (5)	1036.7 (7)
*Z*	2	4	1
μ (mm^−1^)	0.07	0.07	0.61
Crystal size (mm)	0.38 × 0.23 × 0.23	0.48 × 0.40 × 0.35	0.48 × 0.25 × 0.25

Data collection			
No. of measured, independent and observed [*I* > 2σ(*I*)] reflections	3163, 2962, 1920	3349, 3238, 2569	6352, 6046, 5673
*R*_int_	0.029	0.014	0.015
(sin θ/λ)_max_ (Å^−1^)	0.703	0.703	0.704

Refinement			
*R*[*F* ^2^ > 2σ(*F* ^2^)], *wR*(*F* ^2^), *S*	0.058, 0.157, 1.02	0.043, 0.118, 1.04	0.033, 0.087, 1.06
No. of reflections	2962	3238	6046
No. of parameters	345	218	600
No. of restraints	398	0	629
Δρ_max_, Δρ_min_ (e Å^−3^)	0.29, −0.27	0.22, −0.18	0.73, −0.34
Absolute structure	Absolute structure set to match the known *S*-con­figuration at atom C9 of the pyrrolidine residue	Absolute structure chosen arbitrarily	Flack parameter determined by classical intensity fit (Flack & Bernardinelli, 1999 ▸, 2000 ▸)
Absolute structure parameter	−1 (3)	−1.8 (10)	−0.02 (2)

Computer programs: *MSC*/*AFC Diffractometer Control Software* (Molecular Structure Corporation, 1991 ▸), *TEXSAN* *PROCESS* (Molecular Structure Corporation, 1989 ▸), *SHELXT2018* (Sheldrick, 2015*a* ▸), *SHELXL2019* (Sheldrick, 2015*b* ▸), *OLEX2* (Dolomanov *et al.*, 2009 ▸), *publCIF* (Westrip, 2010 ▸) and *PLATON* (Spek, 2020 ▸).

**Table 2:** Selected geometric parameters (Å, °) around the Pd atom of **14**

Pd1—Cl1	2.3049 (19)	Pd1—N1	1.962 (6)
Pd1—Cl2	2.2988 (19)	Pd1—N21	1.999 (5)
			
Cl1—Pd1—Cl2	179.45 (9)	Cl2—Pd1—N1	89.11 (17)
Cl1—Pd1—N1	90.34 (16)	Cl2—Pd1—N21	90.82 (16)
Cl1—Pd1—N21	9.73 (16)	N1—Pd1—N21	179.6 (3)

**Table 3:** Azirine ring geometry (Å, °) in 3-amino-2*H*-azirines

CSD refcode/Compound No.	N1=C3	N1—C2	C2—C3	C3—N4	C3=N1—C2	N1=C3—C2	N1—C2—C3	Reference
ABUKUD	1.271 (3)	1.577 (3)	1.436 (3)	1.333 (3)	59.38 (16)	71.01 (19)	49.61 (15)	Brun *et al.* (2001 ▸)
ABULAK	1.275 (5)	1.577 (5)	1.436 (5)	1.347 (4)	59.4 (2)	70.9 (3)	49.8 (2)	Brun *et al.* (2001 ▸)
ABULEO	1.2712 (13)	1.5766 (16)	1.4290 (16)	1.3401 (12)	59.08 (7)	71.178 (7)	49.74 (7)	Brun *et al.* (2001 ▸)
ABULIS	1.277 (3)	1.570 (3)	1.435 (3)	1.340 (2)	59.49 (14)	70.47 (14)	50.05 (12)	Brun *et al.* (2001 ▸)
ABULOY	1.290 (8)	1.575 (7)	1.442 (10)	1.327 (9)	59.5 (4)	70.1 (5)	50.4 (4)	Brun *et al.* (2001 ▸)
HAGGUR	1.262	1.565	1.454	1.317	60.8	69.9	49.3	Piskunova *et al.* (1993 ▸)
JUNJEH	1.264 (3)	1.565 (3)	1.434 (3)	1.342 (3)	59.8 (2)	70.6 (2)	49.6 (1)	Villalgordo & Heimgartner (1992 ▸)
LERJUN	1.278 (3)	1.568 (3)	1.435 (4)	1.315 (4)	59.55 (18)	70.3 (2)	50.17 (16)	Peters *et al.* (2000 ▸)
MAZRPZ	1.254	1.575	1.428	1.343	59.4	71.6	49.1	Galloy *et al.* (1980 ▸)
PXCAZN	1.279	1.490	1.429	1.317	61.6	66.5	51.9	Galloy *et al.* (1974 ▸)
TIBFUF	1.283 (3)	1.568 (3)	1.438 (3)	1.322 (3)	59.56 (16)	70.12 (16)	50.32 (14)	Bucher & Heimgartner (1996 ▸)
**11**, 2*R* com­ponent	1.280 (6)	1.525 (6)	1.456 (6)	1.323 (5)	61.8 (3)	67.4 (4)	50.8 (3)	This work
**11**, 2*S* major com­ponent	1.281 (6)	1.515 (7)	1.448 (6)	1.323 (5)	61.7 (4)	67.1 (4)	51.1 (3)	This work
**11**, 2*S* minor com­ponent	1.283 (7)	1.517 (7)	1.475 (6)	1.323 (5)	62.9 (4)	66.3 (4)	50.7 (3)	This work
**12**	1.271 (3)	1.588 (3)	1.446 (3)	1.344 (3)	59.55 (14)	71.20 (16)	49.26 (12)	This work
**14** ligand 1	1.294 (8)	1.550 (8)	1.476 (8)	1.326 (8)	61.8 (4)	67.7 (4)	50.5 (4)	This work
**14** ligand 2	1.250 (8)	1.519 (8)	1.465 (9)	1.332 (8)	63.0 (4)	67.5 (5)	49.5 (4)	This work
