# Supplementary material for: Synthesis and crystal structures of new chiral 3-amino-2H-azirines and the Pd com­plex of one of them
Source: Acta Crystallogr C Struct Chem. 2023 Feb 23;79(Pt 3):104–11. doi: 10.1107/S2053229623001468 (PMC9985946; doi:10.1107/S2053229623001468)
Supplement: Supplementary file 7 [file c-79-00104-sup7.pdf]

Supporting Information for article:

Synthesis and crystal structures of new chiral 3-amino-2*H*-azirines and the Pd-complex of one of them

Anthony Linden, Christoph B. Bucher, Ralf Gubler, José M. Villalgordo, and Heinz Heimgartner

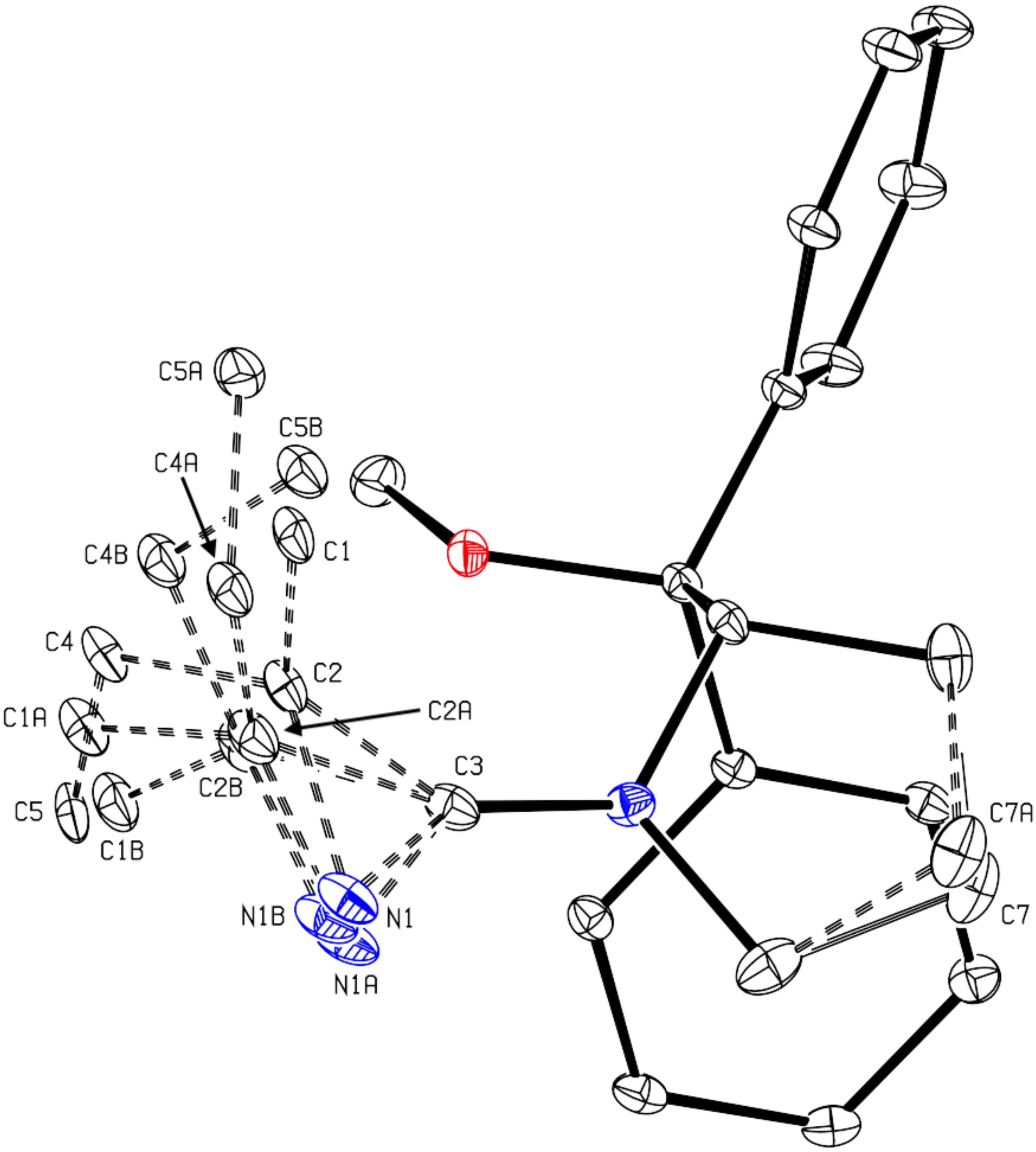

Figure S1 View of the molecular structure of **11** with all disordered components overlaid. Displacement ellipsoids are drawn at the 10% probability level. H atoms have been omitted for clarity.

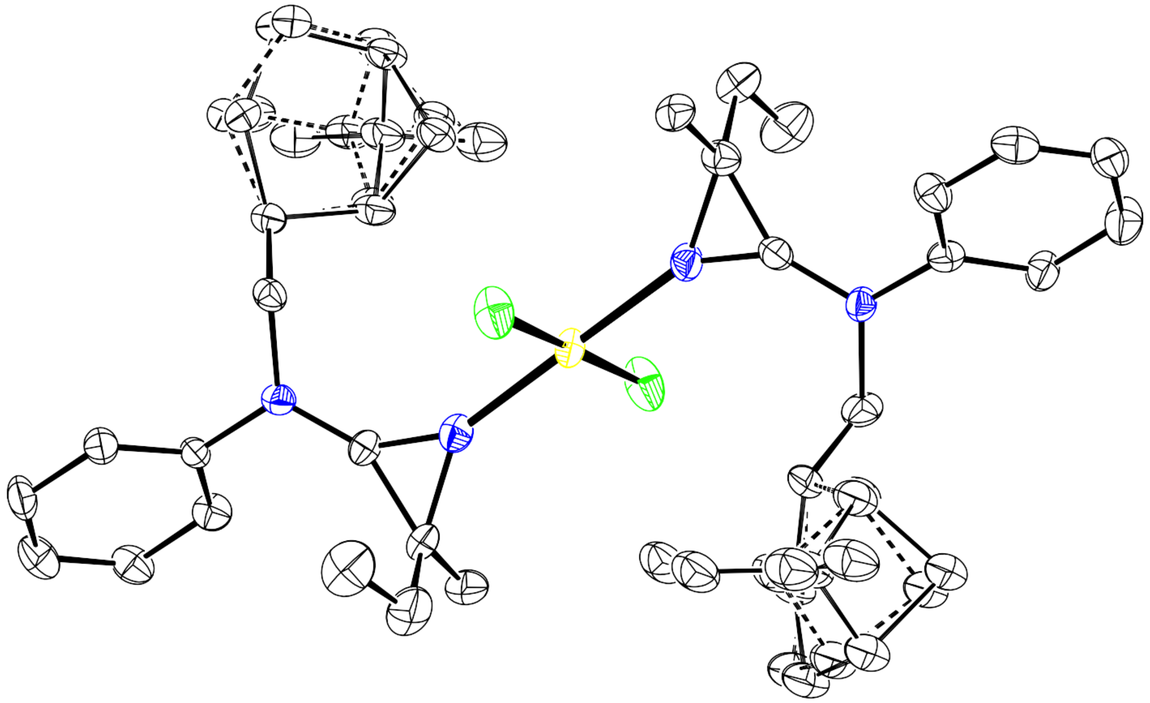

Figure S2 View of the molecular structure of **14** with both disordered components overlaid. Displacement ellipsoids are drawn at the 30% probability level. H atoms have been omitted for clarity.
